# Functional analysis of haplotypes and promoter activity at the 5′ region of the human *GABRB3* gene and associations with schizophrenia

**DOI:** 10.1002/mgg3.652

**Published:** 2019-03-25

**Authors:** Yi Liu, Mei Ding, Yong‐ping Liu, Xi‐cen Zhang, Jia‐xin Xing, Jin‐feng Xuan, Xi Xia, Jun Yao, Bao‐jie Wang

**Affiliations:** ^1^ School of Forensic Medicine China Medical University Shenyang China

**Keywords:** *GABRB3*, haplotype, promoter, schizophrenia, transcriptional regulation

## Abstract

**Background:**

This study investigated the effects of haplotypes T‐G and C‐A derived from NG_012836.1:g.4160T>C and NG_012836.1:g.4326G>A on protein expression levels in vitro and identified the functional sequence in the regulatory region of the *GABRB3 *gene linked to possible associations with schizophrenia.

**Methods:**

Recombinant plasmids with haplotypes T‐G and C‐A and 10 recombinant vectors containing deletion fragments from the *GABRB3 *gene 5′ regulatory region were transfected into HEK‐293, SK‐N‐SH, and SH‐SY5Y cells. The relative fluorescence intensity of the two haplotypes and different sequences was compared using a dual luciferase reporter assay system.

**Results:**

The relative fluorescence intensity of haplotype C‐A was significantly lower than that of T‐G. We shortened the core promoter sequence of the *GABRB3 *gene 5′ regulation region from −177 bp to −18 bp (ATG+1). We also found an expression suppression region from −1,735 bp to −1,638 bp and an enhanced regulatory region from −1,638 bp to −1,335 bp. Multiple inhibitory functional elements were identified in the region from −680 bp to −177 bp.

**Conclusion:**

We demonstrated that haplotype C‐A might increase the risk of schizophrenia and found multiple regulatory regions that had an effect on *GABRB3* receptor expression.

## 1 INTRODUCTION

Schizophrenia is a complex and highly hereditary disease (Sullivan, Kendler, & Neale, 2003). A large number of studies have shown that the γ‐aminobutyric acid neurotransmitter system (GABA) plays an important role in the pathogenesis of schizophrenia. As early as 1999, Wassef et al. discovered that GABAergic compounds could be used to treat schizophrenia (Wassef, Baker, & Kochan, 2003). Other studies have shown that increased activity of the GABAergic system is associated with decreased concentration of magnesium ions in plasma and cells in schizophrenia patients (Nechifor, 2011). *GABA* is an important inhibitive neurotransmitter in the central nervous system. By binding with its receptor, *GABAi* can lead to an imbalance between brain excitation and inhibitory signals and have potentially far‐reaching implications for the maintenance of normal cognition, emotion, and perception (Dienel & Lewis, 2018; Fatemi & Folsom, 2015). Of the many *GABA* receptor subtypes, *GABRB3 *(gamma‐aminobutyric acid type A receptor beta 3, subunit gene, MIM#137192) is one of the candidate genes for studying mental illnesses, and has been a hot spot for recent research (Huang, Cheng, Tsai, Lai, & Chen, 2014).

The gene encoding the* GABRB3* receptor is located on human chromosome 15 and has multiple functional SNPs in its regulatory region. NG_012836.1:g.4160T>C is a polymorphic locus from the transcription initiation site in the 5′ regulatory region of the *GABRB3* gene to upstream 897 bp, and its allele C has been found to be associated with an increased risk of heroin dependence (Chen, Huang, & Liao, 2014). In 2006, Urak et al. successfully constructed four haplotypes by detecting 13 SNP loci in the* GABRB3 *gene 5′ regulatory region and found that one of the haplotypes may be related to the occurrence of remitting childhood absence epilepsy (rCAE). It has also been confirmed that decreased* GABRB3* gene expression may be a potential pathogenic factor in rCAE (Urak, Feucht, Fathi, Hornik, & Fuchs, 2006). Therefore, different haplotypes derived from different SNPs may directly or indirectly affect the expression of *GABRB3* receptor mRNA and protein and further affect *GABRB3 *neurotransmitter function (Hogart, Nagarajan, Patzel, Yasui, & Lasalle, 2007).

There are at least five transcriptional initiation sites in the *GABRB3* gene, which can potentially produce various protein subtypes. Exon 1A is located upstream of the 5′ regulatory region and plays an important role in brain development (Ben‐Ari, Khazipov, Leinekugel, Caillard, & Gaiarsa, 1997; Kirkness & Fraser, 1993). The 230 bp sequence of the *GABRB3* gene from transcriptional initiation to upstream has been identified as the core promoter region, which contains exon 1A (Tanaka et al., 2012). Although current studies have confirmed a strong link between transcription factor‐4 (TCF‐4) and the development of schizophrenia, we know very little about the gene that encodes transcription factor regulation (Xia et al., 2018). Moreover, studying functional fragments of the *GABRB3* gene regulatory region and the potential association with schizophrenia have not been specific enough. The results of research on functional regions and polymorphisms remain controversial.

Our group previously identified an association between NG_012836.1:g.4160T>C and NG_012836.1:g.4326G>A in the 5′ regulatory region of the* GABRB3* gene by Sanger sequencing and hypothesized that the C‐A haplotype may increase the risk of schizophrenia (Liu et al., 2018). Therefore, we further explored the effects of the two haplotypes on gene expression at the protein level in vitro and constructed multiple recombinant vectors containing sequences of different lengths from the *GABRB3* gene promoter region. The functional sequences of the *GABRB3* gene regulatory region and subsequent effects on the* GABRB3* receptor were further investigated to clarify the association between the receptor and schizophrenia.

## 2 MATERIALS AND METHODS

### 2.1 Ethical compliance

The study was approved by the China Medical University Review Committee. All blood samples were collected in accordance with the principle of informed consent.

### 2.2 Construction of pGL‐3 recombinant vectors of two NG_012836.1:g.4160T>C and NG_012836.1:g.4326G>A haplotypes in the 5′ regulatory region of GABRB3

Restricted endonucleases were selected according to the pGL‐3Basic plasmid polyclonal site and Primer 5.0 software design primers. Cleavage sites for Kpn I and Bgl II were introduced into the 5′ end of the primers. Primer sequences were as follows:

Forwards: 5′‐GGGGTACCATGCACGGTTGGATAA‐3′.

Reverse: 5′‐GAAGATCTGTGCCTGCAGAACGCC‐3′.

The reference sequence NG_012836.1 of *GABRB3 *was used.

**Figure 1:**
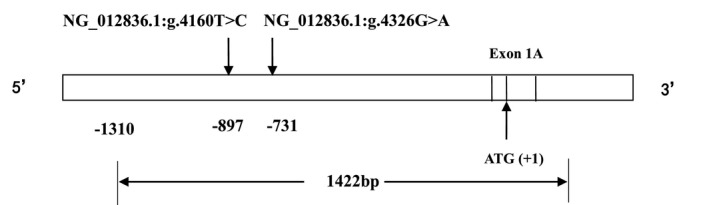
The 1,422 bp cloned fragment spans from −1,310 to +112 in the* GABRB3 *gene, including two SNPs. The GenBank reference sequence from −1,310 to +112 bp (ATG+1) of the* GABRB3* gene is as follows, version number NG_012836.1: 5′‐ATGCACGGTTGGATAATAATGTTTGTAATGTGGCTTTGCAAGTCTTAGAACATATAAACTGGTAGTAAGGGGGCTAATTCTTGCTATGTTGTTGGTTTTATTAAAACAACTTCTTTTCATTGTTTACTTTTTATAGCCACGATCCCCAGCAGGGCATTTCTCCAAAAGAACTGCATGCAAATAGGAGAAATAAACTGAAATATGAATGTGTGTGTGATGCAACAGATATAATTTTCCAGATAATTCCATTGTGCATTAACAGAACATTGAATGCAAAGCAGTGGTGAATAGACTTTTCCAGAAAATAATTAGGATGCTATATTTATCTCTGAATCTTTCAGGTACTGCGGTCACATTTTCTGTTCCAAAAATAAAATAGCTGATTTTTTAAAAAGGCAGTATATTTAAAATTAATGCACAGAAACATGCCAACGTGAGATTTAGGATCTAGATTTAGGTATTTACTAATATAAAGTCTCAACGATGTTAAAAGCTTAAGCTTCATGGGAAAAAAAATGAGTCAATACAGGAAAGTAGTTATCTATTATTAACCATTCATTAAGTCCTGGAATAATCTGAAGAAGTCTACAGAACACAAAAACGAGCTTGATGTGTAGATTTTGTTAGTTGGCTACAAGTAGGATTGTGTAACCATCTTCTTCAATATTAGAAGTCCGAGGTACAGATAAACTTTATTGGGGATCACTCACTAAAATATAAAACCTGTGGCCGTAGGTGAGTGGCCCCTCAGGTGTGCGGTGGTGGTCCAGAGGGTGGGGTGCATCCGGTGTGCACTGGTACACCAGGGTCCTTGCACCAGTGCGCCAGTAGCCTTCTAATGACAGCCGAAGGAGGCCTGCTGCAGGGAAGCAAGGACCCTTGCCTTATATTAAGGACCACGGATAGCTCTGGGCGGCACCAAAAAAGGCACGTATTTTACCTGGAATGACAATCCAGTCTCCAAGTCTGGCTGGGATTCCAGTTTGCTGATAACAAAACACATATTCAGTCCTCACTTAACCATCCATTACATTTGTATATATGTGAGCAGAAAGGGCTCAGTGCTCCCAAGAAATGTTCAGGAGTGAGGGTAAGAGGTGCAGTTTAAGGAGCAGCTGTTAAAAAAAAAAGGTCCAATTGTATAAATGAAAAATAGGGCCGCCACGGCAGGGGCTGGAAGACGGGTCAGGCGGGAAAGCCTGGGGGTGGGGGTGGGGGTAGGGGCGGGGATCCCTGCGTCGCCGTTTGGCTGCTCGGAGAGTAGGGGGGAGAGCGGATCCCAGCAGGTTAGGCCGGAGGAACAGCGCCATGTGCTCCGGGCTCCTGGAGCTCCTGCTGCCCATCTGGCTCTCCTGGACCCTGGGGACCCGAGGCTCTGAGCCCCGCAGGTGAGGCGGGGGCTTCCCGGCGTTCTGCAGGCAC‐3′

According to previous sequencing results and haplotype analysis, DNA samples from haplotypes T‐G and C‐A derived from two SNPs were selected as templates for PCR amplification of the target fragment (Figure 1). The purified target gene was cloned into the pGM‐T vector (TIANGEN, Beijing, China), and the recombinant vector was accurately screened out by transformation and sequencing. The pGM‐T recombinant vector was re‐cloned from the pGL‐3 vector (Promega, Madison, WI), and eukaryotic cells from the two recombinant vectors were transfected (Wu et al., 2017).

### 2.3 Construction of pGL‐3 recombinant vectors for different sequence fragments from the GABRB3 gene 5′ regulatory region

Primers (Table 1) and cleavage sites for the restriction enzymes Kpn I and Bgl II were introduced into the 5′ end to amplify target fragments. In this study, the longest target fragment was located in the* GABRB3 *gene from −1,880 bp to +113 bp (ATG+1)(NG_012836.1). This segment was used as an amplification template to construct 10 truncated fragments with a 5′ deletion. The purified target gene was also cloned into the pGM‐T vector. Through transformation, the recombinant vector of the target fragment was accurately screened by sequencing. The pGM‐T recombinant vector was then cloned into a pGL‐3 vector. Recombined pGL3 vectors were selected in the same manner to perform eukaryotic cell transfection.

**Table 1:** Primer sequences of the target fragments containing the cleavage sites

Target fragments	Primer sequences
Kpn I——GABRB3 (−1,880) F	5′‐GGGGTACCAGGTGACACTTCTAGTG‐3′
Kpn I——GABRB3 (−1,735) F	5′‐GGGGTACCTGGCATGGAAAGAG‐3′
Kpn I——GABRB3 (−1,638) F	5′‐GGGGTACCTCATCTTTGAGAGGC‐3′
Kpn I——GABRB3 (−1,335) F	5′‐GGGGTACCAAGGTGAAGCCAAG‐3′
Kpn I——GABRB3 (−978) F	5′‐GGGGTACCGAATCTTTCAGGTACTG‐3′
Kpn I——GABRB3 (−680) F	5′‐GGGGTACCTGGCTACAAGTAGGATT‐3′
Kpn I——GABRB3 (−587) F	5′‐GGGGTACCACCTGTGGCCGTA‐3′
Kpn I——GABRB3 (−485) F	5′‐GGGGTACCGCCAGTAGCCTT‐3′
Kpn I——GABRB3 (−365) F	5′‐GGGGTACCGAATGACAATCCAG‐3′
Kpn I——GABRB3 (−177) F	5′‐GGGGTACCGTCCAATTGTATAAATG‐3′
Bgl II——GABRB3 (+113) R	5′‐GAAGATCTTGCCTGCAGAACGC‐3′

Note

The primer sequences were shown, and the 5′ end of the primer sequences contained the Kpn I and Bgl II restriction enzyme cleavage sites. The number in brackets was the 5′ end position in the *GABRB3* gene as ATG+1. F = forward, R = reverse. The reference sequence NG_012836.1 of *GABRB3 *was used.

### 2.4 Cell culture

Human embryonic kidney cell line HEK‐293 and human neuroblastoma cell lines SK‐N‐SH and SH‐SY5Y were transfected with recombinant vectors. HEK‐293 cells were cultured in HyClone® DMEM high glucose medium containing 10% fetal bovine serum (Thermo Fisher Scientific, MA), while SK‐N‐SH cells were cultured in KeyGEN BioTECH® DMEM high glucose medium with 0.011 g/L sodium pyruvate containing 15% fetal bovine serum. SH‐SY5Y cells were cultured in HyClone® DMEM/F‐12 mixed medium. Cell transfection was performed when cell density reached 90%.

### 2.5 Transient transfection and dual luciferase reporter assay

Cells were inoculated in 24‐well plates at a density of 2 × 10^5 ^cells per well. After 36–48 hr in culture, recombinant vectors and the pRL‐TK vector (Promega, Madison, WI) were cotransfected with Lipofectamine® 3000 transfection reagent (Invitrogen, CA). After 24 hr, the cell lysate was collected to detect luciferase (LUC) and sea kidney luciferase (TK). In each experiment, recombinant vectors were represented in triplicate and the experiment was repeated three times.

### 2.6 Statistical calculations

LUC/TK was calculated as the relative fluorescence intensity. Each sample is expressed as the mean ± *SD* (standard deviation). The mean comparison of multiple samples was performed by one‐way analysis of variance (ANOVA), while the mean comparison of two samples was performed using the LSD‐T test. *p* < 0.05 represents a statistically significant difference. SPSS 20.0 software was used for statistical analysis.

### 2.7 Transcription factor predictions

Bioinformatics analysis of functional fragments with significant protein expression was performed. JASPAR software (http://jaspar.genereg.net/cgi-bin/jaspar_db.pl) (Krasnov et al., 2016) was used to predict transcription factors. Screening conditions included transcription factors that bound to DNA sequences with at least a >80% match score.

## 3 RESULTS

### 3.1 Construction of a luciferase reporter vector in the GABRB3 gene 5′ regulatory region

**Figure 2:**
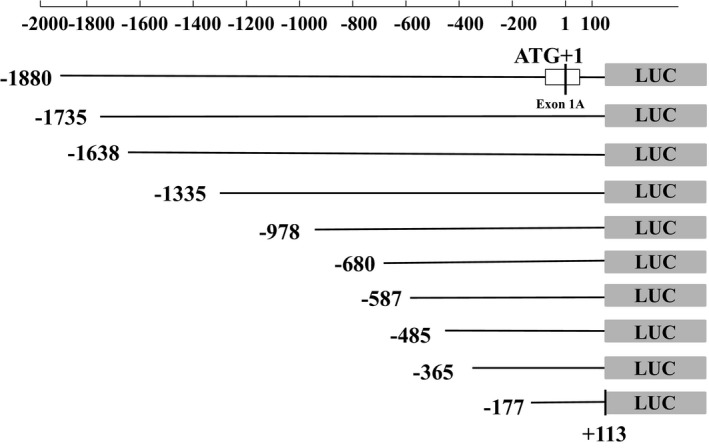
Ten pGL3 recombinant vectors containing different fragments. This figure shows the location of target fragments in the 10 recombinant vectors obtained from the 5′ end of the* GABRB3 *gene regulatory region. The longest target fragment (1993 bp) was located at −1,880 to +113 bp as ATG+1. The 3′ end of the other amplified fragments was unchanged. The GenBank reference sequence from −1,880 to +113 bp as ATG+1 of the* GABRB3* gene is as follows, version number NG_012836.1: 5′‐AGGTGACACTTCTAGTGACTTGCCGACTAATTTACAACTCAGCCTAGGTTCCCCAGAAAACATATCCTGAGGCAAAATTTAGGTGGTGATATGTTACTAGGATGTTTGATTCCAATGAAAACAGGAGCAAAGGAAAAGGGAATTGTGGCATGGAAAGAGAGGGAGCAGATAGGAGGGTTCATTACCAGGCAGACCACCCACTGCTGAGTATGAAGTACAAAAGATGGATCCATCTCACAGGATCATCTTTGAGAGGCCAACAGCGACTGCATCTCAGGACATTTCTTAGGAGGCAATAAAGGGAGACTCTTTGATCCATGGATATGTGTCCATTAGACAAAAGTCTGCCTCATAGACTTCTAACTTGCCTGAAATTTCAGATTGCATATATGTGAGTACTGAGCAAATCCCGTGGCATCTCAGACCTTGCGGATATGAGGTGATCATATCCCATGTGGGCTTGTTCCCATGTAAAGCTGATCAGACCTCTTGCAGAACTGGGTGCTGAGGCAGCAGCTGGGGTGATAAAGCCAAAAGCTCCAGAGAAGGTGAAGCCAAGAAGGTCTGATGTGATGCACGGTTGGATAATAATGTTTGTAATGTGGCTTTGCAAGTCTTAGAACATATAAACTGGTAGTAAGGGGGCTAATTCTTGCTATGTTGTTGGTTTTATTAAAACAACTTCTTTTCATTGTTTACTTTTTATAGCCACGATCCCCAGCAGGGCATTTCTCCAAAAGAACTGCATGCAAATAGGAGAAATAAACTGAAATATGAATGTGTGTGTGATGCAACAGATATAATTTTCCAGATAATTCCATTGTGCATTAACAGAACATTGAATGCAAAGCAGTGGTGAATAGACTTTTCCAGAAAATAATTAGGATGCTATATTTATCTCTGAATCTTTCAGGTACTGCGGTCACATTTTCTGTTCCAAAAATAAAATAGCTGATTTTTTAAAAAGGCAGTATATTTAAAATTAATGCACAGAAACATGCCAACGTGAGATTTAGGATCTAGATTTAGGTATTTACTAATATAAAGTCTCAACGATGTTAAAAGCTTAAGCTTCATGGGAAAAAAAATGAGTCAATACAGGAAAGTAGTTATCTATTATTAACCATTCATTAAGTCCTGGAATAATCTGAAGAAGTCTACAGAACACAAAAACGAGCTTGATGTGTAGATTTTGTTAGTTGGCTACAAGTAGGATTGTGTAACCATCTTCTTCAATATTAGAAGTCCGAGGTACAGATAAACTTTATTGGGGATCACTCACTAAAATATAAAACCTGTGGCCGTAGGTGAGTGGCCCCTCAGGTGTGCGGTGGTGGTCCAGAGGGTGGGGTGCATCCGGTGTGCACTGGTACACCAGGGTCCTTGCACCAGTGCGCCAGTAGCCTTCTAATGACAGCCGAAGGAGGCCTGCTGCAGGGAAGCAAGGACCCTTGCCTTATATTAAGGACCACGGATAGCTCTGGGCGGCACCAAAAAAGGCACGTATTTTACCTGGAATGACAATCCAGTCTCCAAGTCTGGCTGGGATTCCAGTTTGCTGATAACAAAACACATATTCAGTCCTCACTTAACCATCCATTACATTTGTATATATGTGAGCAGAAAGGGCTCAGTGCTCCCAAGAAATGTTCAGGAGTGAGGGTAAGAGGTGCAGTTTAAGGAGCAGCTGTTAAAAAAAAAAGGTCCAATTGTATAAATGAAAAATAGGGCCGCCACGGCAGGGGCTGGAAGACGGGTCAGGCGGGAAAGCCTGGGGGTGGGGGTGGGGGTAGGGGCGGGGATCCCTGCGTCGCCGTTTGGCTGCTCGGAGAGTAGGGGGGAGAGCGGATCCCAGCAGGTTAGGCCGGAGGAACAGCGCCATGTGCTCCGGGCTCCTGGAGCTCCTGCTGCCCATCTGGCTCTCCTGGACCCTGGGGACCCGAGGCTCTGAGCCCCGCAGGTGAGGCGGGGGCTTCCCGGCGTT CTGCAGGCA‐3′

The 1,422 bp target gene for haplotype analysis of the *GABRB3* gene 5′ regulatory region was cloned into a pGL‐3 vector. Recombinant plasmids containing T‐G and C‐A haplotypes derived from NG_012836.1:g.4160T>C and NG_012836.1:g.4326G>A SNPs were successfully screened by sequencing. Ten different *GABRB3* gene sequence lengths were successfully cloned into the pGL‐3 vector. After sequencing, inserted fragments were identical to the primer design fragment sequences. Figure 2 shows details of the target segments of the 10 truncated fragments.

### 3.2 Analysis of relative fluorescence intensity of T‐G and C‐A haplotypes

**Figure 3:**
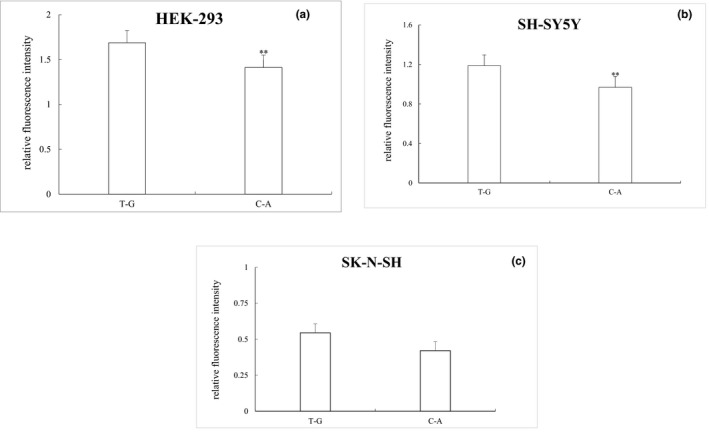
Relative fluorescence intensity of two different haplotypes in HEK‐293, SH‐SY5Y, and SK‐N‐SH cells. The T‐G plasmid was compared with wild type C‐A, which had the highest relative luciferase activity. **p < 0.05

Three cell lines (HEK‐293, SK‐N‐SH, and SH‐SY5Y) were used to report luciferase levels. By comparing the relative fluorescence intensity with pGL‐Control, both haplotypes were shown to express luciferase. The relative fluorescence intensity of the C‐A plasmid in the HEK‐293 cell line was significantly lower than in the T‐G plasmid (*p* = 0.008, Figure 3a). The expression trend was similar in the SH‐SY5Y cell line (*p* = 0.018, Figure 3b). However, there was no significant difference in the relative fluorescence intensity between the two plasmids in the SK‐N‐SH cell line (*p* = 0.15, Figure 3c).

### 3.3 Pairwise comparative analysis of relative fluorescence intensity of 10 recombinant vectors

In this experiment, 10 recombinant vectors with different fragment lengths were transfected into the three cell lines to verify the effect of different positions in the *GABRB3* gene regulatory region on *GABRB3 *protein expression. The target fragment of 290 bp located in (−177 to +113 bp) (ATG+1) had the highest relative fluorescence intensity in HEK‐293, SK‐N‐SH, and SH‐SY5Y cell lines.

**Figure 4:**
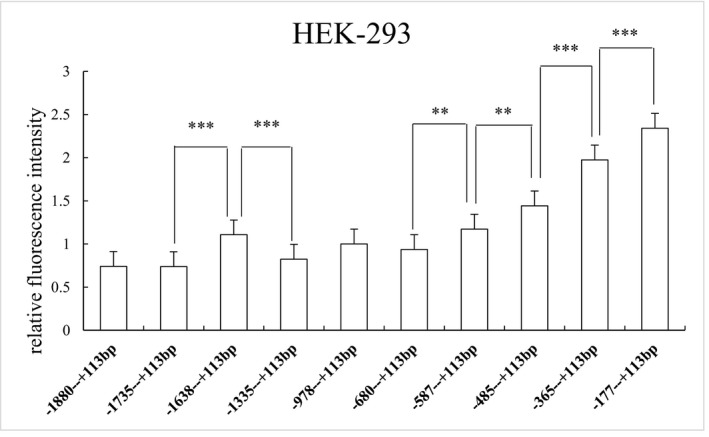
Relative fluorescence intensity of 10 recombinant vectors in HEK‐293 cells. There was a significant difference in relative fluorescence intensity between (−1,735 to +113 bp) versus (−1,638 to +113 bp) and (−1,638 to +113 bp) versus (−1,335 to +113 bp)(ATG+1). There were four regions with significant regulatory effects on gene expression between (−680 to +113 bp) versus (−587 to +113 bp), (−587 to +113 bp) versus (−485 to +113 bp), (−485 to +113 bp) versus (−365 to +113 bp), and (−365 to +113 bp) versus (−177 to +113 bp)(ATG+1). Relative fluorescence intensity is expressed as the mean ± *SD*. Differences in relative fluorescence intensity were determined by the LSD‐T test. ** 0.001 ≤ *p *≤ 0.05, *** *p* < 0.001

Pairwise comparison of 10 truncated fragments obtained from the 5′ terminal was conducted in HEK‐293. There was a significant difference in relative fluorescence intensity between fragments, as follows: (−1,735 to +113 bp) versus (−1,638 to +113 bp), (−1,638 to +113 bp) versus (−1,335 to +113 bp), (−680 to +113 bp) versus (−587 to +113 bp), (−587 to +113 bp) versus (−485 to +113 bp), (−485 to +113 bp) versus (−365 to +113 bp), and (−365 to +113 bp) versus (−177 to +113 bp)(ATG+1). *p* values were 0.000, 0.000, 0.002, 0.001, 0.000, and 0.000, respectively (Figure 4).

**Figure 5:**
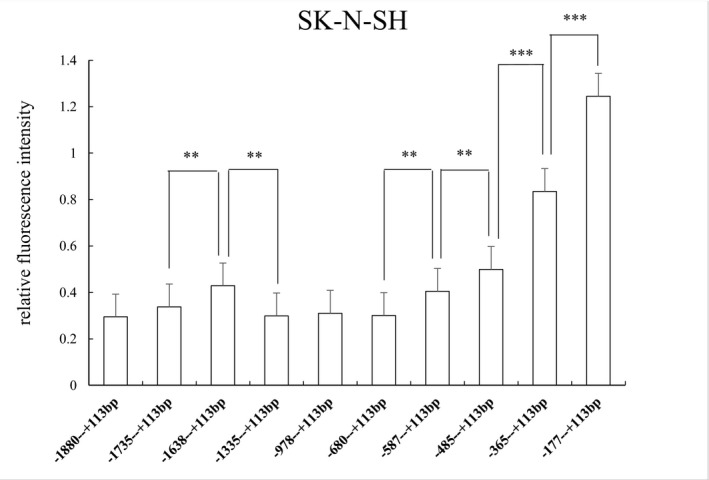
Relative fluorescence intensity of 10 recombinant vectors in SK‐N‐SH cells. There was a significant difference in relative fluorescence intensity between (−1,735 to +113 bp) versus (−1,638 to +113 bp) and (−1,638 to +113 bp) versus (−1,335 to +113 bp)(ATG+1). There were four regions with significant regulatory effects on gene expression between (−680 to +113 bp) versus (−587 to +113 bp), (−587 to +113 bp) versus (−485 to +113 bp), (−485 to +113bp) versus (−365 to +113 bp), and (−365 to +113 bp) versus (−177 to +113 bp)(ATG+1). Relative fluorescence intensity is expressed as the mean ± *SD*. Differences in relative fluorescence intensity were determined by the LSD‐T test. ** 0.001 ≤ *p *≤ 0.05, *** *p* < 0.001

In the SK‐N‐SH cell line, the protein expression trend was similar to the HEK‐293 cell line, although the relative fluorescence intensity was lower. The relative fluorescence intensity was still significantly different between target fragments: (−1,735 to +113 bp) versus (−1,638 to +113 bp), (−1,638 to +113 bp) versus (−1,335 to +113 bp), (−680 to +113 bp) versus(−587 to +113 bp), (−587 to +113 bp) versus (−485 to +113 bp), (−485 to +113 bp) versus (−365 to +113 bp), and (−365 to +113 bp) versus (−177 to +113 bp)(ATG+1). *p* values were 0.019, 0.001, 0.007, 0.014, 0.000, and 0.000, respectively (Figure 5).

**Figure 6:**
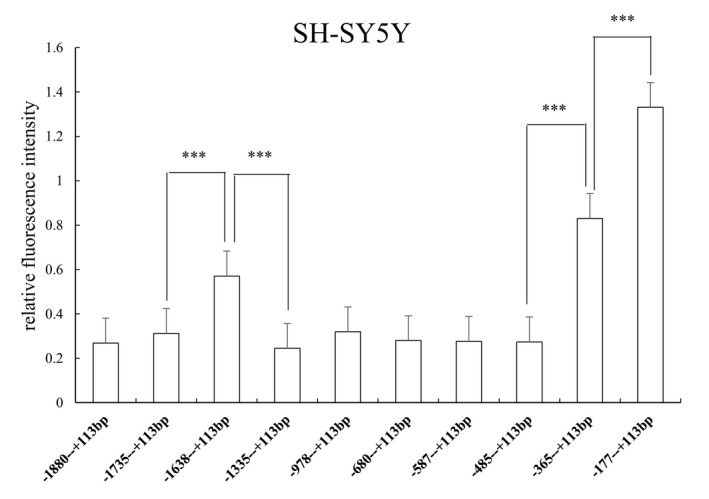
Relative fluorescence intensity of 10 recombinant vectors in SH‐SY5Y cells. There was a significant difference in relative fluorescence intensity between (−1,735 to +113 bp) versus (−1,638 to +113 bp) and (−1,638 to +113 bp) versus (−1,335 to +113 bp)(ATG+1). There were two regions with significant regulatory effects on gene expression between (−485 to +113 bp) versus (−365 to +113 bp) and (−365 to +113 bp) versus (−177 to +113 bp)(ATG+1). Relative fluorescence intensity is expressed as the mean ± *SD*. Differences in relative fluorescence intensity were determined by the LSD‐T test. *** *p* < 0.001

In SH‐SY5Y cell lines, the relative fluorescence intensity of 10 truncated fragments was the lowest. Luciferase expression trends between (−1,735 to +113 bp) versus (−1,638 to +113 bp) and (−1,638 to +113 bp) versus (−1,335 to +113 bp)(ATG+1) were similar to the previous two cell lines, and the relative fluorescence intensity was significantly different (*p* = 0.000). In addition, only (−485 to +113 bp) versus (−365 to +113 bp) and (−365 to +113 bp) versus (−177 to +113 bp)(ATG+1) were found to have significant statistical differences in relative fluorescence intensity (*p* = 0.000, Figure 6).

## 4 DISCUSSION

In this study, 1,422 bp fragments from the *GABRB3* gene 5′ regulatory region were inserted into a pGL‐3 luciferase reporter gene vector and transfected into HEK‐293, SK‐N‐SH, and SH‐SY5Y cells to examine the effects on luciferase protein expression by two haplotypes of T‐G and C‐A derived from NG_012836.1:g.4160T>C and NG_012836.1:g.4326G>A. In comparing the relative fluorescence intensity of T‐G and C‐A plasmids in HEK‐293 and SH‐SY5Y cells, a significant difference was observed between the two plasmids. Luciferase protein expression of the C‐A plasmid was much lower than the T‐G plasmid. Haplotype base composition of the two plasmids suggests that this result may be produced by NG_012836.1:g.4160T>C. NG_012836.1:g.4160T>C is a tag SNP in which the C allele has been linked to the development of multiple psychiatric disorders, such as CAE (Tanaka et al., 2008) and heroin dependence (Chen et al., 2014). These disorders have the same or similar symptoms and behavior as schizophrenia (Berrettini, 2003; Tsuang, Taylor, & Faraone, 2004). The C allele of NG_012836.1:g.4160T>C may increase *GABRB3 *receptor expression, while the *GABRB3 *receptor exerts a rapid inhibitory effect through ligand‐bound cl‐channels (Fatemi, Folsom, & Thuras, 2017), which regulate protein expression, thus enabling luciferase protein expression in the C‐A plasmid to be lower than T‐G. *GABRB3 *protein expression has been confirmed to be low in the cerebellum and BA9 area of schizophrenic patients (Fatemi et al., 2017), which is consistent with the results of this study. However, Chen's group did not find an association between the* GABRB3 *gene and its SNP locus with schizophrenia and autism spectrum disorder. This may be due to different races evaluated in the selected populations, differences in screening the survey population and psychosocial factors that can have an impact on research. In a previous study, we found that NG_012836.1:g.4160T>C and NG_012836.1:g.4326G>A were in linkage disequilibrium, forming haplotypes T‐G and C‐A, and that haplotype C‐A could increase the risk of schizophrenia (Liu et al., 2018). The results of this study further validate previous experimental results at the protein level. Haplotype C‐A may increase *GABA* neurotransmitters by increasing *GABRB3* receptor expression, thereby reducing protein expression. However, the two SNPs did not regulate protein expression in SK‐N‐SH cells. Possible use of undifferentiated SK cells may have contributed to this result. Compared with mature neurons in the human body, undifferentiated cells have different neuron‐specific enzymes, and cannot fully respond to regulation of the *GABRB3* gene promoter region in vivo (Ba, Pang, Davidge, & Benishin, 2004). Different cells have their own specificity and splitting abilities, leading to possible differences in expression of the same gene.

**Figure 7:**
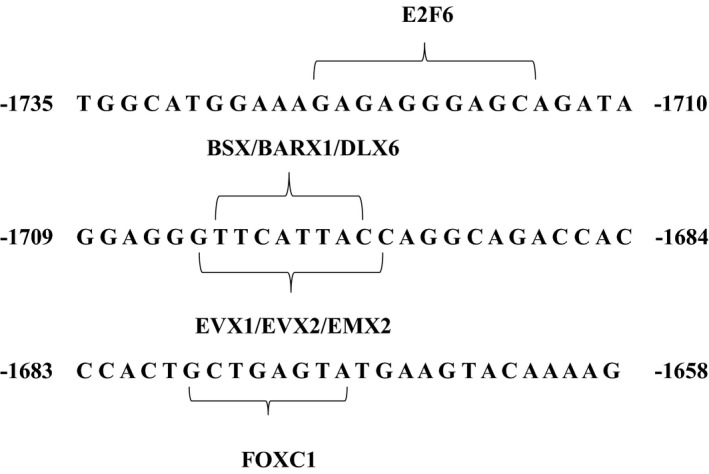
Transcription factors and prediction of functional fragments. Predictive results of transcription factors between −1,735 bp to −1,658 bp (ATG+1) are shown. Numbers before the fragments are at the 5′ end position in the *GABRB3* gene. Bases in parentheses were recognized by transcription factors in which a perfect match was considered 80%

To further study the functional sequence of the 5′ regulatory region in the *GABRB3 *gene, 10 pGL‐3 recombinant vectors with different length fragments were constructed and transfected into three cell lines. Differences in protein expression were analyzed. In HEK‐293, SK‐N‐SH and SH‐SY5Y cell lines, the target fragment located at −177 bp to +113 bp (ATG+1) had the highest relative fluorescence intensity, which might indicate a central transcriptionally active region of the *GABRB3* gene. The −230 bp to −18 bp (ATG+1) sequence in the* GABRB3* gene 5′ regulatory region was previously confirmed as the core promoter region (Tanaka et al., 2012). However, we more precisely identified the core promoter region of the* GABRB3 *gene from −177 bp to −18 bp (ATG+1). Rs20317 is a SNP‐site located in this region. Its C allele regulates transcriptional activity by binding to transcription factors CMYB and EGR‐3, thus promoting protein expression (Tanaka et al., 2012). We performed additional detailed truncation analysis of the promoter region in both neuroblastoma cell lines and HEK cells. In the three cell lines, the relative fluorescence intensity increased significantly when the sequence located between −1,735 bp to −1,638 bp was truncated. When the sequence between −1,638 bp to −1,335 bp (ATG+1) was truncated, protein expression decreased significantly. There may be an inhibitory region in the former sequence and an enhanced regulatory factor in the latter sequence. Through sequence analysis using JASPAR software, many transcription factors, such as E2F6, FOXC1, and EVX1/X2, were identified in the region from −1,735 bp to −1,638 bp (ATG+1) (Figure 7). Transcription factor EVX1/X2 affects the properties of spinal cord neurons and influences spinal neuron neurotransmitters (Juarez‐Morales et al., 2016).

In HEK‐293 and SK‐N‐SH cell lines, we found that luciferase expression activity of (680 to +113 bp), (−587 to +113 bp), (−485 to +113 bp), (−365 to +113 bp), and (−177 to +113 bp)(ATG+1) increased step by step and that stronger relative fluorescence intensity was observed closer to the core promoter region. There may be many regulatory factors in the −680 bp to −177 bp (ATG+1) sequence that can inhibit protein expression activity. In SH‐SY5Y cells, only (−485 to +113 bp), (−365 to +113 bp), and (−177 to +113 bp) (ATG+1) showed significant differences in increased luciferase expression activity. This may be due to differences between species and cell lines or possibly due to region specificity of certain regulatory factors, thereby affecting different regulatory factors in different cell types. Several studies identified the transcription factor binding site RE‐1 in the region from −680 bp to −365 bp (ATG+1) that can bind to the inhibitory transcription factor NRSF (neuron‐restrictive silencer factor) or rest (the repressor element 1‐silencing transcription factor) to inhibit transcriptional activity and likely plays an important role in neuronal epigenetics (Ballas & Mandel, 2005; Chen, Paquette, & Anderson, 1998). However, NG_012836.1:g.4160T>C and NG_012836.1:g.4326G>A are located in sequences from −978 bp to −680 bp (ATG+1), which had no effect on protein expression in the three cell lines. Up‐regulated functional elements in the sequence may attenuate the inhibitory effects of haplotype C‐A, resulting in no significant change in luciferase activity in this sequence.

In this study, the 5′ promoter region of the* GABRB3 *gene was truncated, and possible functional sequences in the* GABRB3* gene were preliminarily identified. However, uncertainty remains regarding the regulatory factors and functional elements that actually play a role in each region. Although transcriptional factors have been predicted in some regions, further experiments are needed to verify whether these transcription factors bind and affect protein expression.

In conclusion, using luciferase reporter gene experiments, we further demonstrated that the haplotype C‐A derived from NG_012836.1:g.4160T>C and NG_012836.1:g.4326G>A may increase the risk of schizophrenia at the level of protein expression in vitro. By constructing pGL‐3 recombinant vectors with different length fragments from the *GABRB3* gene regulatory region, we showed a shortened core promoter sequence from −177 bp to −18 bp (ATG+1). We found an expression suppression region from −1,735 bp to −1,638 bp and an enhanced regulatory region from −1,638 bp to −1,335 bp (ATG+1). Multiple inhibitory functional elements in the region from −680 bp to −177 bp (ATG+1) were also identified. However, transcription factor binding sites, transcription factors, and other regulatory factors in the *GABRB3* gene still need to be identified in future research.

## 5 CONFLICT OF INTEREST

The authors declare no conflict of interests.
